# Extended Poststroke Rehabilitation Combined with Cerebrolysin Promotes Upper Limb Motor Recovery in Early Subacute Phase of Rehabilitation: A Randomized Clinical Study

**DOI:** 10.3390/medicina59020291

**Published:** 2023-02-03

**Authors:** Sindi Z. Mitrović, Ljubica M. Konstantinović, Vera Miler Jerković, Suzana Dedijer-Dujović, Olivera C. Djordjević

**Affiliations:** 1 Clinic for Rehabilitation “Dr. Miroslav Zotović”, Faculty of Medicine, University of Belgrade, Sokobanjska 13, 11000 Belgrade, Serbia; 2 Innovation Center, School of Electrical Engineering, University of Belgrade, Bulevar Kralja Aleksandra 73, 11120 Belgrade, Serbia

**Keywords:** stroke, severe impairment, subacute stage, Cerebrolysin, upper limb motor recovery, extended rehabilitation

## Abstract

*Background and Objectives*: The recovery of stroke patients with severe impairment is usually poor and limited and, unfortunately, under-investigated in clinical studies. In order to support neuroplasticity and modulate motor recovery, Cerebrolysin combined with rehabilitation treatment has proven effective in the acute stroke phase in moderate to severe motor impairment. The aim of this study was to determine the efficacy of extended poststroke rehabilitation combined with Cerebrolysin on upper limb motor recovery in subacute stroke patients with severe upper limb motor impairment. *Materials and Methods*: A randomized, double-blind, placebo-controlled study was conducted. Sixty patients at the early stage of severe sub-acute stroke who fulfilled all eligibility criteria were randomly assigned to the Cerebrolysin group or placebo group (𝑛 = 30 each). Both groups, after conducting three weeks of conventional rehabilitation treatment five days per week, continued to perform conventional rehabilitation treatment three times per week until 90 days of rehabilitation treatment. The primary outcome measure was the Action Research Arm Test (ARAT), and the secondary outcomes were the Fugl-Meyer Assessment-Upper Extremity (FMA-UE) motor score, Barthel index (BI), and the National Institutes of Health Stroke Scale (NIHSS). The outcome data were evaluated before, after three weeks of treatment, and on the 90th day of rehabilitation treatment, and compared within groups and between the two groups. There were no adverse events. *Results*: Both groups showed a significant improvement (*p* < 0.001) over time in BI, FMA-UE, ARAT, and NIHSS scores. Patients receiving Cerebrolysin showed more significant improvement in post-stroke upper limb motor impairment and functioning compared to the placebo group after only three weeks, and the trend was maintained after 90 days of follow up. *Conclusion*: Cerebrolysin delivered in the early subacute post-stroke phase added to extended conventional rehabilitation treatment is beneficial and improves motor functional recovery in patients with severe motor impairment, especially on the paretic upper extremity.

## 1. Introduction

Stroke is a neurological disease with the highest degree of acquired disability worldwide [1]. Despite receiving therapy, 25–30% of people who survive a stroke remain unable to independently carry out the various activities of daily living [2]. The process of reversing the motor deficit of an upper limb is more complex and time-consuming than the recovery of a lower limb. Furthermore, following neurorehabilitation, only 5–20% of stroke patients achieve complete functional recovery of the upper limb [3], while walking function is not regained in approximately 35% of the patients with impaired motor function of the lower limb [4]. In addition to the motor deficit, the presence of comorbidities, the impairment of emotional and psychological functions, and cognition contribute to a significantly lower quality of life in the post-stroke period [5,6].

The disability encountered after stroke and recovery is individual, with significant variability, which renders post-stroke rehabilitation management globally challenging [7]. New therapeutic approaches in stroke rehabilitation are primarily focused on neuroplasticity and motor learning, with the idea that a positive clinical effect is achieved by restoring motor function through intensive and repetitive task-specific training. Evidence suggests that neurological recovery through brain reorganization or through compensation may occur in the subacute and chronic stages following stroke [8,9]. Regardless, the most significant recovery is expected within the first three months following the stroke [10] when neuroplasticity is significantly higher and spontaneous recovery is more likely [11].

Regarding the recovery of upper limb motor function after stroke, even after six months of rehabilitation, 60–70% of stroke patients still have a motor impairment that causes disability and affects the activities of daily living [12,13]. Namely, for optimal arm functioning in patients, the presence of rough mobility of the limb is insufficient without coordination and the ability to perform fine movements [14]. Despite all selective movements being performable in proximal segments, an optimal clinically significant degree of arm functioning will still not be achieved [15]. Consequently, the improvements in functional outcomes of the upper limb require long-term, multidisciplinary treatment and rehabilitation to achieve functional independence and improve the quality of life.

In recent years, a new pragmatic therapeutic approach was considered to achieve maximum recovery in optimum time in treating stroke, known as “recovery enhancers” [16]. In addition to therapeutic rehabilitation interventions, the simultaneous application of some pharmacological agents with neurorestorative abilities is recommended to enhance the beneficial effects of neurorehabilitation on motor impairment. Cerebrolysin (EVER Neuro Pharma GmbH, Austria) is one of these neuroprotective agents whose multimodal effects on motor recovery was demonstrated first in animal models and recently in clinical studies. It is a low-molecular-weight neuropeptide preparation obtained through the standardized enzymatic proteolysis of porcine brain proteins. As a potent neuropeptide, it affects neurotrophy and neuroprotection, especially concerning neuronal recovery through the activity of the neurotrophic factor (NTF) and sonic hedgehog (Shh) signaling pathways [17,18]. Accordingly, it has the potential to amplify the capacity of neuroplasticity in early subacute post-stroke rehabilitation through synaptic remodeling and transmission, reduction in neurovascular reconstruction, neurite outgrowth, and oligodendrogenesis [16,19]. Furthermore, it is essential to point out that Cerebrolysin can be used without a time window limitation for the drug administration and relevant restrictions [16]. According to evidence from several randomized controlled trials conducted on the safety and adverse effects of Cerebrolysin in acute and early subacute stroke rehabilitation, it has shown a good safety profile, with no significant changes in vital and laboratory parameters and without sufficient evidence in terms of risk of mortality compared to placebo-treated patients [20,21]. Moreover, in various clinical guidelines with class III recommendation, Cerebrolysin has been recommended as part of the pharmacological support in acute and subacute motor rehabilitation after ischemic stroke [22,23,24,25].

Most clinical studies have shown the synergistic effect of the combination of Cerebrolysin and conventional therapy on motor recovery in the acute stroke phase in moderate to severe motor impairment [22,26,27,28,29,30,31,32]. Only a few studies have dealt with the subacute stroke phase and severe motor impairment, focusing on upper limb recovery [33,34,35].

This study aimed to determine the efficacy of extended poststroke upper limb rehabilitation combined with Cerebrolysin on motor functional recovery in subacute stroke patients with severe upper limb motor impairment. The primary outcome measure was the Action Research Arm Test (ARAT), as it values functional recovery of the arm, and the secondary outcomes were the Fugl-Meyer Assessment-Upper Extremity (FMA-UE) motor score and the Barthel index (BI).

## 2. Materials and Methods

The double-blind, randomized, placebo-control research was conducted at the Clinic for Rehabilitation “Dr. Miroslav Zotovic” in Belgrade over three years from March 2019 to March 2022. The design of this study was the double-blind because neither patients nor the data collectors know which treatment the patients were receiving until the study was over. The study included 110 patients of both sexes of at least 18 years of age with neuroradiologically confirmed ischemic stroke and severe motor impairment (National Institutes of Health Stroke Scale (NIHSS) >14) [36]. The patients were referred to the neurorehabilitation inpatient department of the Clinic for Rehabilitation “Dr. Miroslav Zotovic” in Belgrade between the seventh and the fourteenth day following the stroke.

The criteria for inclusion of patients in the study were the following: (a) the first stroke in a subacute phase (7–14 day lapse from the insult); (b) stroke of cortical or subcortical localization verified by computerized tomography or nuclear magnetic resonance imaging; (c) patients with unilateral paresis of the upper extremities of a severe degree (FME-UE ≤ 25) [37]; (d) balance of the trunk allowing a seated position; and (e) patients with the ability to comprehend and follow simple instructions (The Mini-Mental State Examination (MMSE) > 24) [38]. The criteria for exclusion of patients from the study included: (a) repeated strokes; (b) patients with bilateral paresis; (c) a previous record of psychiatric illnesses or other neurological diseases; (d) pregnancy or lactation; (e) medical conditions that might significantly influence the understanding and performance of the foreseen therapeutic protocol (severe cardiovascular diseases, kidney diseases, liver diseases, severe cognitive and perceptive impairment, contractures/contraction of upper extremities); (f) previous application of other brain peptides; (g) allergies to Cerebrolysin or conditions in which the use of Cerebrolysin is contraindicated; and (h) severe deficits in memory that impede proper measurement performance. Due to the COVID-19 pandemic, from March 2020, an excluding factor was also active SARS-CoV-2 infection in the past three months before inclusion in the study.

Of a total of 112 patients assessed for eligibility, 60 patients met the criteria and entered the study. All of the examined patients were inpatients undergoing rehabilitation treatment, and all were subjected to thorough anamnesis, laboratory, and clinical evaluation. The total number of patients excluded from the study was 52. The number of participants who failed to meet the inclusion criteria was 27. The number of those excluded due to comorbidities that made it impossible to carry out the treatment (frequent oscillations of blood pressure, dizziness in an upright position, or febrile conditions) was 13. Twelve patients declined to participate.

All participants signed an informed consent that allowed for their participation in the research, which the Clinic’s Ethics Committee approved (No. 03-1518).

### 2.1. Sample Size

To determine the needed sample size, a two-tailed independent *t*-test with a 0.05 tolerance of reporting a false positive (α) and an 80% probability of not reporting a false negative (power) was used. The effect size was set as 0.20 (improvement in FMA-UE from pre-time to post-time) with a standard deviation of 0.27 [39]. In the pilot study, which included five subjects, the ARAT parameter was tested in the pre- and post-time points. The mean value with standard deviation in time point ‘pre’ was 4.8 (3.6), while in time point ‘post’ it was 8.1 (4.3). The Cohen’s effect size was 0.8. According to sample size calculations based on a paired *t*-test using library pwr (RStudio), with a test power of 95% and a significance level of 5%, we determined that the desired sample size was 23.

### 2.2. Interventions

The patients were allocated randomly into two groups using 60 sequentially numbered, opaque, sealed envelopes that had been prepared earlier using a computerized table of random numbers (using RSudio), and which were balanced to ensure equal numbers in each group. The allocations were concealed from the statistician until the statistical analysis had been completed. Significant heterogeneity exists amongst the studies regarding time of treatment initiation, treatment dosage, and duration. In our study, the dosage of Cerebrolysin was based on previously published data from a meta-analysis of nine randomized trials evaluating Cerebrolysin’s clinical efficacy compared to a placebo. Treatment efficacy was demonstrated primarily with daily 30 mL Cerebrolysin infusions for 10 or 21 days when initiated within 12 to 72 h of symptom onset [30]. Moreover, Chang et al. reported that such a daily dose of Cerebrolysin (30 mL) during the three-week administration had a beneficial effect on motor recovery in patients at the early stage of severe sub-acute stroke comparable to placebo [33]. The Cerebrolysin group (*n* = 30) received 30 mL of Cerebrolysin through an intravenous line placed on the non-paretic arm, diluted in 70 mL normal saline (total infusion solution 100 mL) over a time period of 30 min once a day for 21 days. The placebo group (*n* = 30) received 100 mL of saline (instead of Cerebrolisyin) through an intravenous line placed on the non-paretic arm over a time period of 30 min once a day for 21 days. Subjects from both groups received conventional inpatient rehabilitation treatment five days per week for three weeks. After 21 days of conventional inpatient rehabilitation treatment, the patients in both groups continued to perform conventional outpatient rehabilitation treatment three times a week until 90 days from the start of the rehabilitation. After 21 days of conducting an inpatient rehabilitation protocol daily, the patients and their families were given instructions for self-management and activities of daily life (ADLs) on the non-therapeutic days. The daily program of conventional rehabilitation consists of 45 to 60 min of physiotherapy and 45–60 min of occupational therapy, depending on the patient’s endurance. Initially, we planned to record the duration of the therapy as well. Regardless, the patients showed daily variations in endurance, and we recorded only the minimum and maximum duration of the treatment. Occupational therapy for the paretic/plegic upper limb included instructions and training towards self-care tasks and ADLs. Physiotherapy included passive stretching within sub-maximal ranges of motion to inhibit spasticity; active assisted movements; range of motion (ROM) exercises for upper and lower extremities; the facilitation of active voluntary movement; exercises to improve endurance, balance, strength, and gait; and other functional tasks assigned depending on patient progress. Between physiotherapy and occupational therapy, the patients had a break lasting 45 min to 1 h. If necessary, the conventional rehabilitation program also included speech therapy three times per week during inpatient and outpatient rehabilitation for both groups.

### 2.3. Safety Criteria

The rehabilitation protocol was carried out individually for all patients under the supervision of a physio and occupational therapist with a graduate degree and vast experience in neurological rehabilitation. Clinicians had performed laboratory tests and medical evaluations at the beginning of the study, and at least once a week during inpatient rehabilitation, as well as at the end of the study (90 days from start of the rehabilitation). In addition, all subjects were asked about any new symptoms in order to identify the adverse effects of treatment.

### 2.4. Outcome Measures

Stroke severity was recorded using the NIHSS through 15 items on the levels of consciousness, language, neglect, visual-field loss, extraocular movement, motor strength, ataxia, dysarthria, and sensory loss. Items were scored on a 3- to 5-point scale, with a maximum total score of 42 points. According to the NIHSS, stroke severity levels may be stratified as follows: very severe: >25; severe: 15–24; mild to moderately severe: 5–14; mild: 1–5 [40].

The primary outcome for evaluating upper limb recovery was the Action Research Arm Test (ARAT), as it values functional recovery of the arm through coordination, dexterity, and upper extremity function. The subject is seated upright in a chair with a firm back and no armrests for the test’s administration [40]. The test consists of 19 functional tasks divided into four domains (grasp, grip, pinch, and movement of the whole hand) arranged in order of decreasing difficulty. According to Lyle, developer of the test, items are scored using a four-point ordinal scale from 0 (no movement) to 3 (movement performed normally) with a maximum total score of 57 points [41]. Considering that the assessment with the ARAT test is based on the continuous monitoring and mobility of the patient, there are no cut-off scores. In stroke rehabilitation, the test can be used to predict the functional recovery of the upper extremity: scores of less than 10 points correlate with poor recovery, between 10 and 56 points correlate with moderate recovery, and scores of 57 points and higher predict good recovery [42].

Secondary outcomes were the Fugl-Meyer assessment score (FMA) and the Barthel Index (BI). The FMA is used to assess upper extremity motor impairment. A widely used FMA subscale for the assessment of motor function is the FMA-UE motor score, which assesses voluntary and synergistic movements of the upper extremity through evaluating the upper extremity as a whole, in addition to wrist, hand and coordination/speed. The body function test is scored on a three-point ordinal scale, and item scores are summed to provide a maximum score of 66 [41,43].

The BI assesses ADL. It includes ten items of ADL, and all items are rated based on the amount of assistance required to complete each activity. The items bathing and grooming are scored 0 or 5; the items feeding, dressing, controlling the bladder, controlling the bowel, getting on and off the toilet, and ascending and descending stairs are scored as 0, 5, or 10. Items for transferring from a wheelchair to the bed and walking on a level surface are scored as 0, 5, 10, or 15. The maximum score is 100, which corresponds to complete independence, and the minimum score is 0, corresponding to total dependence [44]. The BI is an activity scale for ADL that is easy to use and shows reliable scoring improvement during the rehabilitation of patients with severe neurological, neuromuscular, and musculoskeletal diseases [45].

All outcomes were evaluated at baseline (start of the treatment), after the intervention (immediately after the treatment on day 21), and on the 90th day of rehabilitation treatment by a physiotherapist experienced in the rehabilitation of neurological disease patients who were blinded to group assignment.

### 2.5. Statistical Analysis

To test for the effects of Cerebrolysin across all time points (pre-therapy, post-therapy, and after 90 days), we used a two-way repeated measures analysis of variance (ANOVA) with time and groups as the within-patient factor and the between-patient factor, respectively, when the conditions were fulfilled. Otherwise, a robust two-way repeated measures analysis of variance with time as a group factor was used. The generalized Eta-Squared was used as a measure of effect size, with an interpretation small closer value of 0 and a large closer value of 1.

The one-way repeated ANOVA was used when the conditions were fulfilled to test the changes in the outcome measures over time within each group separately. Otherwise, the Friedman test was used. Within ANOVA, multi-comparisons were conducted using a paired *t*-test. Within the Friedman test, multi-comparisons were performed using the Wilcoxon test. Bonferroni correction was used to correct the effect of multiple comparisons.

The assumption of sphericity was tested by the Mauchly’s test. In cases when the assumption of sphericity has been violated, the Sphericity corrections were used. The Greenhouse–Greisser (GG) and the Huynh–Feldt (HF) were used for adjusting the degrees of freedom from the repeated measures ANOVA. If the GG value was greater than 0.75, the HF value was used.

The assumptions related to approximately normally distributed data at each time point were tested using the Shapiro–Wilk test and normal QQ plots.

The independent *t*-test, or the Mann–Whitney test, was used to compare continuous outcomes between the two groups. The Chi-Squared test or Fisher’s exact test were performed on categorical outcomes. In addition, the adequate effect size was calculated for tests. The Cohen effect size was calculated for the independent *t*-test and paired *t*-test, with interpretations of small (0.20), medium (0.50), and large (0.80). The Wilcoxon effect size for the Mann–Whitney test and the Wilcoxon test yielded small (0.10–0.30), medium (0.30–0.50), and large (>0.50) interpretations. The Cramer’s *V* effect size was calculated during the testing of the categorical outcomes, with small (0.10–0.30), medium (0.30–0.50), and large (>0.50) interpretations.

A statistical analysis was conducted in RStudio (version 1.4.1106). The basic level of statistical significance was set at 0.05.

## 3. Results

**Figure 1:**
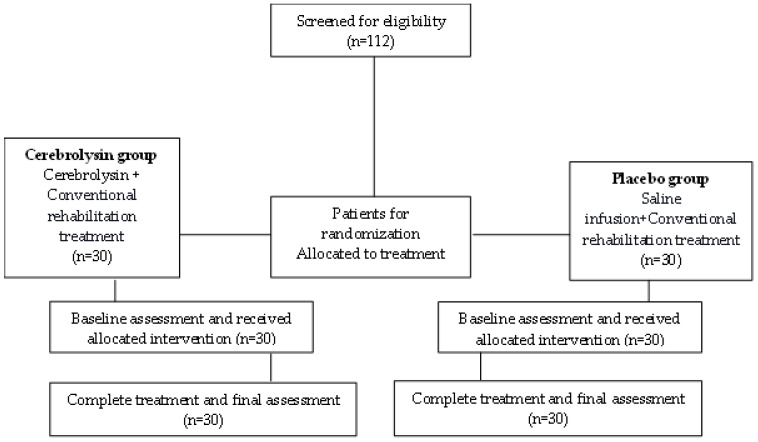
Flow diagram of patient recruitment.

One hundred and twelve patients with severe post-stroke hemiparesis were screened for eligibility criteria. Sixty patients fulfilled all eligibility criteria. Twenty-seven did not meet the inclusion criteria, thirteen patients were medically unstable, and twelve declined to participate in the clinical trial. Figure 1 shows a flow diagram of patient recruitment throughout the study.

There were no significant differences in baseline demographic and clinical parameters between groups. Descriptive statistics of all outcomes in both groups are presented in Table 1.

Thirty participants were included in each group. There was no statistically significant difference in sex (*p* = 0.152), age (*p* = 0.454), family status (*p* = 0.225), education (*p* = 0.943), or in duration (*p* = 0.436) between participants of the Cerebrolysin group and participants of the placebo group. In addition, there is no statistically significant difference in baseline characteristics before any therapy in stroke side (*p* = 0.721), stroke lesion (*p* = 0.523), MMSE (*p* = 0.381), NIHSS (*p* = 0.987), BI (*p* = 0.623), FMA-UE (*p* = 0.509), or in ARAT (*p* = 0.278).

**Figure 2:**
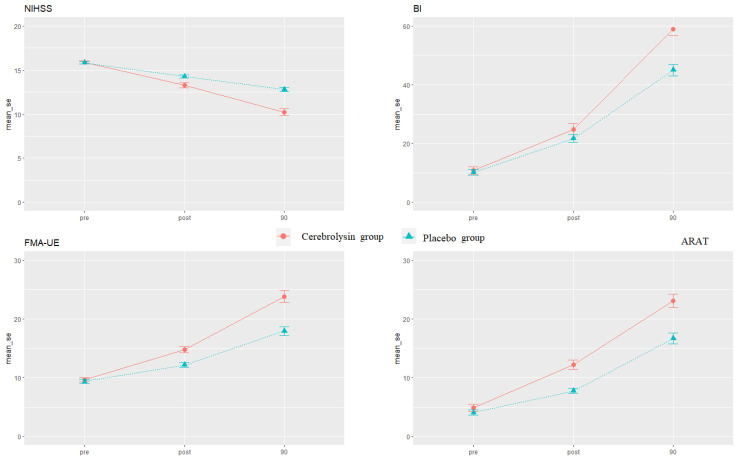
The interaction effect between time and therapy in NIHSS, FMA-UE, BI, and ARAT. The Y-axis represents mean values with error bars. The X-axis represents time points pre, post, and after 90 days.

The results of testing the changes in the outcome measures (NIHSS, BI, FMA-UE, and ARAT) over time are presented in Table 2 and Figure 2.

There is a statistically significant two-way interaction between the type of therapy and time for all outcomes (NIHSS: F (2.58) = 56.1, *p* < 0.0001; BI: F (2.58) = 24.3, *p* < 0.0001; FMA-UE: F_H_(1.6, 47.6) = 27.6, *p* < 0.0001; ARAT: F_G_(1.6, 46.2) = 17.6, *p* < 0.0001).

**Figure 3:**
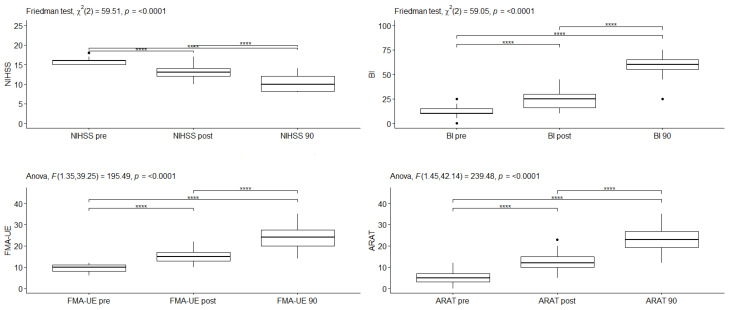
The changes in the outcome measures over time for NIHSS, BI, FMA-UE and ARAT in the Cerebrolysin group. **** *p* < 0.0001.

The changes in outcome measures over time at each level of treatment are statistically significant in all outcomes for the Cerebrolysin group (Figure 3 and Table 3) (NIHSS: χ^2^(2) = 59.5, *p* < 0.0001;BI: χ^2^(2) = 59.05, *p* < 0.0001; FMA-UE: F_G_(1.35, 39.25) = 195.5, *p* < 0.0001; ARAT: F_G_(1.45, 42.14) = 239.5, *p* < 0.0001).

**Figure 4:**
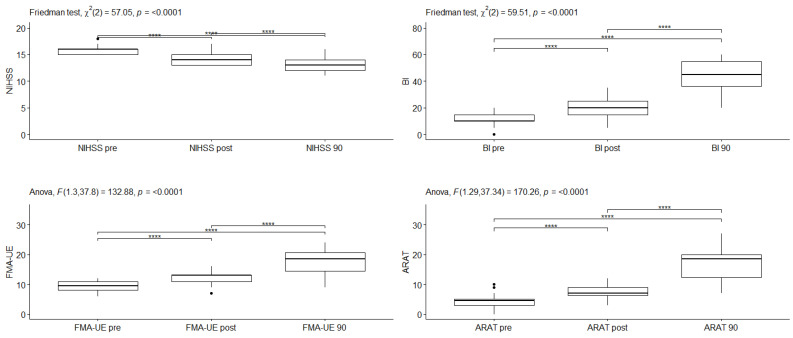
The changes in outcome measures over time for NIHSS, BI, FMA-UE, and ARAT in the Placebo group. The solid circles represent the outliers (without effect). **** *p* < 0.0001.

The changes in the outcome measures over time at each level of treatment are statistically significant in all outcomes (Figure 3 and Table 3 for the Cerebrolysin group and Figure 4 and Table 4 for the placebo group).

At different time points, all tested parameters differed significantly in the Cerebrolysin group. The BI, FMA-UE, and ARAT were statistically significantly higher (BI: < 0.0001; FMA-UE: *p* < 0.0001; ARAT: *p* < 0.0001) at the different time points, and all *p* the pairwise differences were statistically significant with a large effect size (BI: W = 0.824; FMA-UE: η^2^ = 0.714; ARAT: η^2^ = 0.710). In all points of follow up, the NIHSS scores were significantly lower and all the pairwise differences were statistically significant, with a large effect size (*p* < 0.0001, W = 0.680).

The changes in the outcome measures over time at each level of treatment were statistically significant in all outcomes for the placebo group (Figure 4 and Table 4) (NIHSS: χ^2^(2) = 57.05, *p* < 0.0001; BI: χ^2^(2) = 59.5, *p* < 0.0001; FMA-UE: F_G_(1.3, 37.8) = 132.9, *p* < 0.0001; ARAT: F_G_(1.29, 37.34) = 170.3, *p* < 0.0001).

At different time points, all tested parameters differed significantly in the placebo group. BI, FMA-UE, and ARAT were statistically significantly higher (BI: *p* < 0.0001; FMA-UE: *p* < 0.0001; ARAT: *p* < 0.0001) at the different time points, and all the pairwise differences were statistically significant with a large effect size (BI: W = 0.768; FMA-UE: η^2^ = 0.598; ARAT: η^2^ = 0.70). In all points of follow up, the NIHSS scores were significantly lower, and all the pairwise differences were statistically significant with a large effect size (*p* < 0.0001, W = 0.559).

Both groups showed a significant improvement over time in the BI, FMA-UE, ARAT, and NIHSS scores. However, the participants receiving Cerebrolysin therapy made more significant improvement than participants in the placebo group (Figure 2).

The significant improvement between two time points in all outcomes of both groups, separately, is presented in Table 5.

Statistically significant differences were shown between the Cerebrolysin and placebo groups in all tested parameters at different time points (NIHSS: pre–post: *p* = 0.0001, w = 0.54; pre-90: *p* < 0.0001, w = 0.80; post-90: *p* < 0.001, w = 0.71; BI: pre-90: *p* < 0.0001, w = 0.74; post-90: *p* < 0.001, w = 0.65; FMA-UE: pre–post: *p* < 0.0001, Cohen’s d = 1.2; pre-90: *p* < 0.0001, Cohen’s d = 1.3, post-90: *p* = 0.001, Cohen’s d = 0.86; ARAT: pre–post: *p* < 0.0001, Cohen’s d = 0.9; pre-90: *p* = 0.0001, Cohen’s d = 1.0). There was no statistically significant difference in ARAT post-90 values.

**Figure 5:**
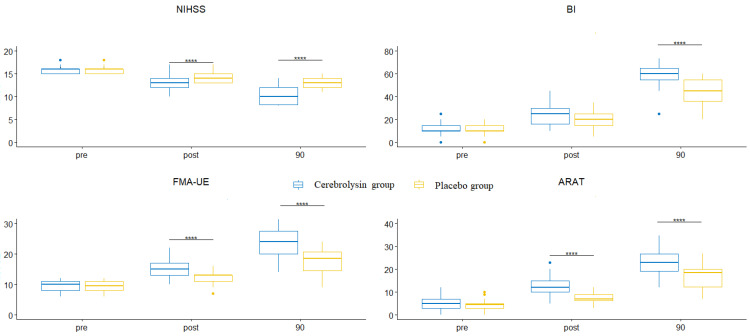
The comparisons in time points between groups in NIHSS, BI, FMA-UE and ARAT. **** *p* < 0.0001.

The comparisons in time points between groups in NIHSS score, Barthel index, FMA-UE and ARAT are presented in Table 6 and Figure 5.

There is no statistically significant difference between groups in the Barthel Index in time point post. All other comparisons between groups are statistically significant with a large effect size, except the NIHSS score with a medium effect size.

No adverse events were registered in either group during or after the application or until the end of the study. No participant dropped out of the study. 

## 4. Discussion

The present study aimed to investigate the effects of a three-week intravenous administration of Cerebrolysin added to the conventional rehabilitation treatment of patients at the early stage of severe sub-acute stroke compared to rehabilitation treatment only. This study’s results have shown that adding Cerebrolysin reduced paretic upper extremity motor impairment and improved functional ability better than rehabilitation treatment alone.

We included patients with severe motor impairment in the early subacute post-stroke phase. Based on animal models and human studies, the data suggest that the subacute stroke stage is characterized by heightened plasticity and neural reorganization, and presents the most critical period for modification by rehabilitation treatment [11,46]. Furthermore, some studies identified that early intensive rehabilitation based on the repetition of specific motor tasks does not have positive effects on the functional recovery of patients in the acute phase, while this therapeutic approach improves neurologic results in the subacute stroke phase [47,48,49]. Neuroplasticity spontaneously increases in early post-stroke rehabilitation, but in patients with severe motor impairment, it tends to reach a plateau after three or six months [11]. Advances in the functional outcome that occurs within three months of the stroke mainly depend on the adaptation strategies of motor learning [18]. In this regard, as additional therapies are added to conventional rehabilitation strategies, numerous therapies for modifying the recovery pattern of post-stroke patients have been considered: task-oriented and repetitive training-based interventions, electromagnetic stimulations, and device-based therapies [50]. However, even with multimodal therapeutic strategies, recovery is usually poor and limited in stroke patients with severe impairment; and unfortunately, these patients remain the least investigated in clinical studies. In order to support neuroplasticity and modulate motor recovery in acute and early subacute stroke phases, many clinical trials of neuropharmacological agents have been conducted. During this period, diminished perilesional inhibition of Gamma-Aminobutyric acid (GABA) and enhanced glutamatergic transmission is important for brain remodeling and motor learning [51]. Preclinical studies and clinical trials provide evidence that neuromodulators such as dopamine agonists, amphetamine-like agents, and serotonin reuptake inhibitors (SSRIs) may increase neuroplasticity [52]. The potential mechanisms of dopamine agonists on sensorimotor function improvement are through the potentiation of drive and arousal responses in conditioned learning and the up-regulation of glutaminergic transmission, which modulates synaptic efficacy [53,54]. However, a recently conducted multicentric randomized trial has found no evidence of the effectiveness of dopaminergic therapy in conjunction with motor therapy on improving walking ability after stroke [55]. Amphetamine-like agents lead to subsequent long-term neuronal reorganization, and norepinephrine has been implicated in trophic changes in the central nervous system [56] and synaptic plasticity that may encode learning [57]. The results from trials with humans concerning the effectiveness of amphetamine-like drugs on motor recovery improvement and ADL in stroke rehabilitation are inconclusive [58]. However, some of them suggest that amphetamine-like drugs only affect upper extremity motor recovery [59] and can improve ADL [60]. SSRIs are widely used in the field of the neuromodulation because, by increasing the availability of this neurotransmitter in the synaptic cleft, they enhance signal transmission [61,62,63] and, consequently, increase the excitatory input of glutamate, activate the NMDA receptors, and lead to synapse reprogramming and strengthening [63]. Kalbouneh HM et al., in their recently updated systematic review, show that SSRIs are effective in preventing and treating depression and improving anxiety, motor function, cognitive function, and dependence in patients after stroke. The authors pointed out that citalopram but not fluoxetine improved the recovery outcomes of patients after stroke, but improvement in the disability was not recorded after treatment with SSRIs. Most studies to date have reported minimal side effects in relation of dopamine agonists and SSRI use in the stroke population [64,65], but special caution is suggested for administrating amphetamine-like drugs in the long term [66]. Considering the variety of plastic mechanisms that enhance motor recovery, the most important role of neuropharmacological agents should be the capacity to simultaneously promote neuroprotection and an ability to switch to neuroplasticity at the same time [67]. Cerebrolysin is one of these multimodal drugs that is available for clinical use. It has a pronounced effect for patients with severe neurological damage during the early post-stroke subacute phase if administered in conjunction with conventional rehabilitation therapy [33,34]. Our results are in line with data from several previous rehab trials that reported on the beneficial effects of Cerebrolysin on motor function in patients with higher baseline stroke severity [31,33,34,68].

In our study, the Cerebrolysin group showed a 2.6 (±1.1)-point improvement in NIHSS score after the therapy and a 5.6 (±1.5)-point improvement in the mean NIHSS score at 90-day follow-up; the placebo group only showed 1.6 (±0.7)-point improvement after the therapy and a 3.0 (±0.8)-point improvement after the 90-day follow-up. Additionally, a meta-analysis of nine randomized trials by Bornstein NM and colleagues demonstrated the treatment efficacy of Cerbolysin compared to placebo through changes in NIHSS greater than four points or the resolution of symptoms [30]. Patients included in the present study had a higher baseline stroke severity than all studies conducted so far, and the Cerebrolysin group had a change of more than four points in the mean NIHSS score after extended rehabilitation treatment. In our study, patients receiving Cerebrolysin showed significantly more improvement based on the findings reported in post-stroke upper limb motor impairment compared to the placebo group after only three weeks, as measured by the FMA-UE, and the trend was maintained after 90 days of follow up. Similarly, the results of our study have also shown the significant improvement in ARAT score to be a measure of functional activities of the upper limb. Higher scores of motor and functional improvement of the upper extremities in the group treated with Cerebrolysin after therapy and after 90-day follow-up indicate positive clinical effects. The effect of Cerebrolysin could be explained by its multimodal impact on the mechanisms of immediate neuroprotection and long-term neuroregeneration enhanced by extended neurorehabilitation [19]. It is worth mentioning that our subjects were in the early subacute post-stroke period with severe upper limb disability. The initial scores of upper limb motor performance measured by FMA-UE were between 7 and 12, and functional activities measured by ARAT were between 0 and 12 in both groups. The subjects’ severe initial impairment and low motor skills in this study indicated a poor prognosis for recovery [37,42]. According to the literature, maximum arm function is achieved by 80% of patients within three weeks after stroke and by 95% of patients within nine weeks of the post-stroke period [11]. The functional impairment of the upper extremity at baseline after stroke is the strongest predictor of motor outcome three months after the stroke [69]. Accordingly, we did not expect the results to be positively correlated with improvements at the clinically significant functional level due to the high level of disability. Nonetheless, it is encouraging that a third of patients in the Cerebrolysin group had an FMA-UE score >25 at 90-day follow-up compared to the placebo group, where no patient reached a score ≥25. These patients in the Cerebrolysin group switched off from severe to moderate to severe impairment of UE and improved their functionality. Consistent with this, motor recovery in the placebo group was also significant but less pronounced than in the Cerebrolysin group. Such results can be explained by the fact that the placebo group also carried out a rehabilitation program during the first three months after the stroke, the most critical therapeutic time window for functional recovery [70]. Furthermore, significant superiority in BI score was noted in the Cerebrolysin group compared to the placebo at the 90-day evaluation. These results reinforce the data found in the previous outcome measures of the study. In addition, compared to the placebo, most patients in the Cerebrolysin group (63.33%) reached the borderline of severe to moderate dependence in everyday functioning. Such a result reflects better global physical abilities, which depend on the recovery of many other functions, to carry out daily activities. It also implies the benefits of more extended rehabilitation treatments for patients with greater motor impairments to fulfill functional training goals in everyday activities and to achieve functional independence, improving quality of life.

However, some clinical studies have reported mixed evidence of Cerebrolysin’s benefit in improving recovery in acute ischemic stroke and safety characteristics. A recent meta-analysis [71] reported that no statistically significant result was detected for Cerebrolysin in the analysis of modified Rankin Scale (mRS), BI and safety outcomes compared with placebo, indicating that Cerebrolysin appears to be safe but of little benefit for acute stroke patients. The overall effect of cerebrolysin on NIHSS was inconsistent among the included clinical studies. Three studies in the meta-analysis showed that Cerebrolysin might be beneficial in improving NIHSS scores, while two studies showed a neutral effect. An updated meta-analysis of seven randomized control trials was published by Ziganshina LE et al. [21] on using Cerebrolysin in acute stroke (first 48 h), reporting with moderate certainty that Cerebrolysin does not reduce the risk of death but increases the number of non-fatal side effects. The beneficial effect confirmed in this study can be explained by the methodological differences between our study and those included in the meta-analyses, primarily manifested in using Cerebroilysin in the acute phase in patients with more severe impairments. Methodological differences are also present in the applied dose and selected parameters for monitoring the effects of therapy. However, the most important thing seems to be that the duration of the rehabilitation treatment was the longest in our study.

### Study Limitations

Several limitations should be considered when interpreting the results of this study. We did not consider factors that can affect the rehabilitation outcome, such as the side and localization of the lesion, motor network plasticity using functional neuroimaging, the presence of comorbidities, or the patient’s cognitive and social status. Another limitation of the study is that the results refer to patients with strict criteria for entering the study regarding disease severity. The duration of rehabilitation treatment for an individual patient is not registered but is given as an approximate minimum and maximum time. A limitation of the study is that no adverse effects were monitored after 90 days. The quality of life assessment was not considered in the current experimental design despite a longitudinal assessment of motor outcome measures after the infusion of Cerebrolysin. Furthermore, no direct assessment of the neurological outcomes, nor paretic versus non-paretic limb performance, was incorporated in the current experimental design.

## 5. Conclusions

Cerebrolysin delivered in the early subacute post-stroke phase added to extended conventional rehabilitation treatment is beneficial and improves motor functional recovery in patients with severe motor impairment, especially on the paretic upper extremity. The three week intravenous administration of Cerebrolysin has shown a good safety profile. Cerebrolysin treatment could be an effective pharmacological supplement to subacute motor rehabilitation after severe ischemic stroke.

## Figures and Tables

**Table 1:** Descriptive statistics of basic characteristics.

Parameters		Cerebrolysin Gorup (*n* = 30)	Placebo Group (*n* = 30)	*p* Value
Age (years) ^1^		55.7 ± 11.2	57.5 ± 11.2	0.454
Sex ^2^	Male	24 (80%)	19 (63.3%)	0.152
	Female	6 (20%)	11 (36.7%)	
Family status ^2^	Single	6 (20%)	6 (20%)	0.225
	Married	24 (80%)	20 (66.7%)	
	Divorced	0 (0%)	4 (13.3%)	
Education ^2^	Primary school	6 (20%)	6 (20%)	0.943
	Secondary school	19 (63.3%)	18 (60%)	
	University	5 (16.7%)	6 (20%)	
Stroke side ^2^	Right	12 (40%)	14 (46.6%)	0.721
	Left	18 (60%)	16 (53.4%)	
Stroke lesion ^2^	Cortical	4 (13.3%)	3 (10%)	0.523
	Subcortical	3 (10%)	5 (16.7%)	
	Cortical-subcortical	23 (76.7%)	22 (73.3%)	
Duration since stroke (days) ^1^		9.77 ± 1.25	9.5 ± 1.1	0.436
MMSE ^1^		25.6 ± 1.45	25.83 ± 1.37	0.381
NIHSS ^1^		15.87 ± 1.0	15.83 ± 0.92	0.987
BI ^1^		10.83 ± 6.95	10.17 ± 4.9	0.626
FMA-UE ^1^		9.67 ± 1.95	9.37 ± 1.89	0.509
ARAT ^1^		4.9 ± 3.36	4.07 ± 2.47	0.279

Legend: MMSE—The Mini-Mental State Examination, NIHSS—National Institute of Health Stroke Scale, BI—Barthel Index, FMA-UE—the Fugl-Meyer assessment score for upper limb, ARAT—The Action Research Arm Test, ^1^ Mean ± Standard Deviation, ^2^ Frequency (Percentage).

**Table 2:** The changes in the outcome measures over time.

	NIHSS	BI	FMA-UE	ARAT
Group	F (1.29) = 37.3	F (1.29) = 24.8	F (1.29) = 43.9	F (1.29) = 48.1
	*p* < 0.0001	*p* =0.00003	*p* < 0.0001	*p* < 0.0001
	η^2^ = 0.158	η^2^ = 0.104	η^2^ = 0.162	η^2^ = 0.183
Time	F (1.5, 42.8) = 418.5	F (2, 58) = 683.8	F (1.2, 33.9) = 252.8	F (1.2, 35.7) = 333.5
	*p* < 0.0001	*p* < 0.0001	*p* < 0.0001	*p* < 0.0001
	η^2^ = 0.628	η^2^ = 0.800	η^2^ = 0.666	η^2^ = 0.702
Group time	F (2, 58) = 56.1	F (2, 58) = 24.3	F (1.6, 47.6) = 27.6	F (1.6, 46.2) = 17.6
	*p* < 0.0001	*p* < 0.0001	*p* < 0.0001	*p* < 0.0001
	η^2^ = 0.134	η^2^ = 0.098	η^2^ = 0.106	η^2^ = 0.073

Legend: NIHSS—National Institute of Health Stroke Scale, BI—Barthel Index, FMA-UE—the Fugl-Meyer assessment score for upper limb, ARAT—The Action Research Arm Test, F—test statistics for two-way repeated measures analysis of variance or robust two-way repeated measures analysis of variance, *p*—*p* value, η^2^—the eta squared effect size.

**Table 3:** The changes in the outcome measures over time for NIHSS, BI, FMA-UE and ARAT for the Cerebrolysin group.

Parameters	Cerebrolysin Group (*n* = 30)	Pre-Post	Pre-90	Post-90	*p* Value ^2^	Effect Size ^2^
		*p* Value ^1^ Effect Size ^1^	*p* Value ^1^ Effect Size ^1^	*p* Value ^1^ Effect Size ^1^		
NIHSS pre	15.87 ± 1.0	<0.0001 0.882 ^w^	<0.0001 0.880 ^w^	<0.0001 0.880 ^w^	<0.0001	0.680 ^kw^
NIHSS post	13.27 ± 1.59					
NIHSS 90	10.23 ± 2.05					
BI pre	10.83 ± 6.95	<0.0001 0.874 ^w^	<0.0001 0.879 ^w^	<0.0001 0.879 ^w^	<0.0001	0.824 ^kw^
BI post	24.83 ± 9.96					
BI 90	58.83 ± 11.04					
FMA-UE pre	9.67 ± 1.95	<0.0001 0.877 ^d^	<0.0001 0.874 ^d^	<0.0001 0.874 ^d^	<0.0001	0.714 ^η2^
FMA-UE post	14.77 ± 2.9					
FMA-UE 90	23.83 ± 5.51					
ARAT pre	4.9 ± 3.36	<0.0001 0.873 ^d^	<0.0001 0.874 ^d^	<0.0001 0.876 ^d^	<0.0001	0.710 ^η2^
ARAT post	12.23 ± 4.5					
ARAT 90	23.13 ± 6.3					

Legend: NIHSS—National Institute of Health Stroke Scale, BI—Barthel Index, FMA-UE—the Fugl-Meyer assessment score for upper limb, ARAT—The Action Research Arm Test, ^1^—*p* value and effect size of the paired *t*-test/the Wilcoxon test, ^2^—*p*-value and effect size of one-way repeated ANOVA/the Friedman test, ^w^—the Wilcoxon effect size, ^d^—The Cohen effect size, ^kw^—the Kendall’s W effect size, ^η2^—the eta squared effect size.

**Table 4:** The changes in outcome measures over time for NIHSS, BI, FMA-UE, and ARAT in the placebo group.

Parameters	Placebo Group (*n* = 30)	Pre-Post	Pre-90	Post-90	*p* Value ^2^	Effect Size ^2^
		*p* Value ^1^ Effect Size ^1^	*p* Value ^1^ Effect Size ^1^	*p* Value^1^ Effect Size ^1^		
NIHSS pre	15.83 ± 0.92	<0.0001 0.866 ^w^	<0.0001 0.865 ^w^	<0.0001 0.867 ^w^	<0.0001	0.559 ^kw^
NIHSS post	14.27 ± 1.08					
NIHSS 90	12.8 ± 1.32					
BI pre	10.17 ± 4.9	<0.0001 0.868 ^w^	<0.0001 0.867 ^w^	<0.0001 0.860 ^w^	<0.0001	0.768 ^kw^
BI post	21.67 ± 7.47					
BI 90	45 ± 10.7					
FMA-UE pre	9.37 ± 1.89	<0.0001 0.856 ^d^	<0.0001 0.854 ^d^	<0.0001 0.853 ^d^	<0.0001	0.598 ^kw^
FMA-UE post	12.17 ± 2.23					
FMA-UE 90	17.9 ± 4.25					
ARAT pre	4.07 ± 2.47	<0.0001 0.835 ^d^	<0.0001 0.834 ^d^	<0.0001 0.834 ^d^	<0.0001	0.70 ^η2^
ARAT post	7.73 ± 2.08					
ARAT 90	16.67 ± 5.06					

Legend: NIHSS—National Institute of Health Stroke Scale, BI—Barthel Index, FMA-UE—the Fugl-Meyer assessment score for upper limb, ARAT—The Action Research Arm Test, ^1^—*p* value and effect size of the paired *t*-test/the Wilcoxon test, ^2^—*p*-value and effect size of one-way repeated ANOVA/the Friedman test, ^w^—the Wilcoxon effect size, ^d^—The Cohen effect size, ^kw^—the Kendall’s W effect size, ^η2^—the eta squared effect size.

**Table 5:** Changes in outcomes over time in the Cerebrolysin group and Placebo group.

Group	Time Points	NIHSS	BI	FMA-UE	ARAT
Cerebrolysin	Pre-post	2.6 ± 1.1	−14 ± 8.7	−5.1 ± 2.3	−7.3 ± 3.3
	Pre-90	5.6 ± 1.5	−48 ± 9.8	−14.2 ± 4.8	−18.2 ± 5.7
	Post-90	3 ± 1.1	−34 ± 8.8	−9.1 ± 4.3	−10.9 ± 4.3
Placebo	Pre-post	1.6 ± 0.7	−11.5 ± 5.9	−2.8 ± 1.5	−3.7 ± 2.2
	Pre-90	3 ± 0.8	−34.8 ± 8.5	−8.5 ± 3.5	−12.6 ± 4.9
	Post-90	1.5 ± 0.8	−23.3 ± 7.7	−5.8 ± 3.3	−8.9 ± 3.9

Legend: NIHSS—National Institute of Health Stroke Scale, BI—Barthel Index, FMA-UE—the Fugl-Meyer assessment score for upper limb, ARAT—The Action Research Arm Test, Mean ± Standard Deviation.

**Table 6:** The comparisons in outcomes between groups.

Parameters	Cerebrolysin Group (*n* = 30)	Placebo Group (*n* = 30)	*p* Value	Effect Size
NIHSS post	13.27 ± 1.59	14.27 ± 1.08	0.006	0.41 ^w^
NIHSS 90	10.23 ± 2.05	12.8 ± 1.32	<0.0001	0.67 ^w^
BI post	24.83 ± 9.96	21.67 ± 7.47	0.168	--
BI 90	58.83 ± 11.04	45 ± 10.7	<0.0001	0.66 ^w^
FMA-UE post	14.77 ± 2.9	12.17 ± 2.23	0.0003	1.01 ^d^
FMA-UE 90	23.83 ± 5.51	17.9 ± 4.25	<0.001	1.2 ^d^
ARAT post	12.23 ± 4.5	7.73 ± 2.08	<0.0001	1.28 ^d^
ARAT 90	23.13 ± 6.3	16.67 ± 5.06	<0.0001	1.13 ^d^

Legend: NIHSS—National Institute of Health Stroke Scale, BI—Barthel Index, FMA-UE—the Fugl-Meyer assessment score for upper limb, ARAT—The Action Research Arm Test, ^d^—The Cohen effect size; ^w^—The Wilcoxon effect size.

## Data Availability

The data that support the findings of this study can be found in the medical system of the Clinic for rehabilitation “Dr. Miroslav Zotović” and are available from the corresponding author upon reasonable request.
